# Methods Used in Economic Evaluations of Chronic Kidney Disease Testing — A Systematic Review

**DOI:** 10.1371/journal.pone.0140063

**Published:** 2015-10-14

**Authors:** Andrew J. Sutton, Katie Breheny, Jon Deeks, Kamlesh Khunti, Claire Sharpe, Ryan S. Ottridge, Paul E. Stevens, Paul Cockwell, Philp A. Kalra, Edmund J. Lamb, eGFR-C study group

**Affiliations:** 1 Health Economics Unit, University of Birmingham, Birmingham, United Kingdom; 2 School of Health and Population Sciences, University of Birmingham, Birmingham, United Kingdom; 3 University of Leicester, Leicester, United Kingdom; 4 King’s College London, London, United Kingdom; 5 Birmingham Clinical Trials Unit, School of Cancer Sciences, Robert Aitken Institute, University of Birmingham, Birmingham, United Kingdom; 6 Kent Kidney Care Centre, East Kent Hospitals University NHS Foundation Trust, Canterbury, Kent, United Kingdom; 7 Queen Elizabeth Hospital Birmingham, Division of Immunity and Infection, University of Birmingham, Birmingham, United Kingdom; 8 Department of Renal Medicine, Salford Royal NHS Foundation Trust, Salford, United Kingdom; 9 Clinical Biochemistry, East Kent Hospitals University NHS Foundation Trust, Canterbury Kent, United Kingdom; Kaohsiung Medical University HospitalKaohsiung Medical University HospitalKaohsiung Medical University Hospital, TAIWAN

## Abstract

**Background:**

The prevalence of chronic kidney disease (CKD) is high in general populations around the world. Targeted testing and screening for CKD are often conducted to help identify individuals that may benefit from treatment to ameliorate or prevent their disease progression.

**Aims:**

This systematic review examines the methods used in economic evaluations of testing and screening in CKD, with a particular focus on whether test accuracy has been considered, and how analysis has incorporated issues that may be important to the patient, such as the impact of testing on quality of life and the costs they incur.

**Methods:**

Articles that described model-based economic evaluations of patient testing interventions focused on CKD were identified through the searching of electronic databases and the hand searching of the bibliographies of the included studies.

**Results:**

The initial electronic searches identified 2,671 papers of which 21 were included in the final review. Eighteen studies focused on proteinuria, three evaluated glomerular filtration rate testing and one included both tests. The full impact of inaccurate test results was frequently not considered in economic evaluations in this setting as a societal perspective was rarely adopted. The impact of false positive tests on patients in terms of the costs incurred in re-attending for repeat testing, and the anxiety associated with a positive test was almost always overlooked. In one study where the impact of a false positive test on patient quality of life was examined in sensitivity analysis, it had a significant impact on the conclusions drawn from the model.

**Conclusion:**

Future economic evaluations of kidney function testing should examine testing and monitoring pathways from the perspective of patients, to ensure that issues that are important to patients, such as the possibility of inaccurate test results, are properly considered in the analysis.

## Introduction

The prevalence of chronic kidney disease (CKD) is high in general populations globally [1–7]. For example, data from the Health Survey in England 2009 and 2010 found the prevalence of low excretory kidney function (glomerular filtration rate [GFR] <60 mL/min/1.73 m^2^) to be 5.2% [8]. The accepted international definition of CKD is abnormalities of kidney structure or function present for ≥3 months, with implications for health [9]. This definition essentially comprises either low excretory kidney function and/or the presence of kidney damage. GFR is recognised ‘as the best overall measure of kidney function, and is frequently used in the diagnosis, staging, and management of CKD’ [10]. In the absence of other evidence of kidney damage, CKD is defined by a GFR threshold of <60 ml/min/1.73m^2^ [11]. Excretory kidney function is commonly assessed using estimates of the GFR (eGFR) derived from serum creatinine measurements and using equations that take account of age, gender, and ethnicity. Detection of albuminuria (increased urinary losses of albumin, including lower amounts historically referred to as microalbuminuria) is also used to identify kidney disease as it indicates the presence of kidney damage irrespective of whether there is also low excretory kidney function [12]. Urinary reagent strip (‘dipstick’) testing is commonly used to screen patients for albuminuria. In order to lessen or remove the impact of kidney failure and lower the risk of cardiovascular disease (CVD) events, patients from both general and high-risk populations such as those with diabetes and hypertension may be screened using eGFR and albuminuria testing with the goal of identifying high risk patients eligible for management to slow the progression of CKD and lower the risk of CVD events [13].

In CKD screening and monitoring are the most common approaches to medical testing. Screening targets patients that are apparently healthy and seeks to identify early disease or emerging risk factors. Monitoring involves using a test repeatedly at various points in time, targeting patients that already have disease e.g. patients with CKD <60 ml/min/1.73m^2^, hypertension, diabetes, etc., to identify any changes in their disease status that would warrant new treatment or a change in treatment regime.

However screening for CKD in the general population may have a negative impact on patients. Although previous studies have suggested that positive CKD tests do not cause psychological harm to patients [14–16], there is still the burden on the patient of having to receive further confirmatory testing, referral to secondary care, and potentially unnecessary treatment [17]. From the perspective of the health care provider there are the costs incurred due to additional patient visits and extra testing, all of which could be spent on other services [18]. Indeed, one of the key issues with CKD is that because the progression of disease is relatively low compared to the variability of the measure used to monitor progression, namely change in GFR or increase in albuminuria, there are likely to be many false positive test results [12].

During the implementation of a testing and diagnostic pathway, delays can occur which prevent patients accessing the optimum treatment pathway for their condition. For example, if testing is delayed then patients that have asymptomatic disease will not get access to the treatment they need, which may lead to unnecessary progression of disease. Then once patients have been tested, delays may occur while waiting for a definitive test result. Furthermore, once the need for treatment has been established, there may be delays accessing this treatment. All of these delays may have an impact on the quality of life of patients. In all cases there is the possibility of the unnecessary progression of disease, while waiting for confirmatory testing and then treatment may lead to unnecessary anxiety. Unnecessary delays may also occur as a result of inefficient health care systems or may be the result of inaccurate testing. For example, a false negative test may give patients false assurance about their health status but result in unnecessary disease progression, potentially leading to increased costs for the health care provider as a result of having to treat more advanced disease later, which is typically more expensive. Even in people with non-progressive CKD the situation is further complicated by the increased likelihood of significant associated complications such as cardiovascular disease events, acute kidney injury, infections, hospitalisations and all cause mortality [19].

An economic evaluation is a comparative study that examines the difference between costs and benefits between two or more options and is used in the estimation of cost effectiveness. There are three main types of economic evaluation: (1) the cost-effectiveness analysis (CEA) which uses an outcome measure in natural units, e.g. cost per case detected, or cost per patient treated; (2) the cost benefit analysis (CBA) which uses an outcome measure in monetary units; and (3) the cost-utility analysis (CUA) which uses the quality adjusted life year (QALY) as the outcome measure. One QALY is defined as one year of life lived in perfect health, and is often the preferred outcome measure in economic evaluations due to its comparability across disease areas. An economic evaluation is conducted from a specific perspective that indicates which costs should be included in the analysis. A health provider perspective means that only the costs incurred by the health care provider will be considered in the analysis, while a societal perspective expands on this perspective to include wider societal costs including the costs incurred by patients, such as travel time and loss of income [20].

Trial based economic evaluations just take cost and outcome data directly from a trial and use these to inform the cost-effectiveness of an intervention over the time horizon of the trial. This approach benefits from being able to take advantage of well designed studies to ensure that the conclusions drawn from the analysis are unbiased. However it is limited to drawing conclusions from only one source of data which may not be representative of the wider population from which it is drawn, and is limited to conducting analysis over the time horizon of the trial, which may not be appropriate for chronic diseases. In contrast, model-based economic evaluations allow the synthesis of data from different sources and allow analysis to continue beyond the end of a trial. This latter approach is more relevant to patients and health care providers who have to consider the impact of these diseases over many years [20]. Ideally the time horizon adopted for a chronic disease should be life-time to ensure that all the patient costs and outcomes are contained within the analysis. However the model-based approach does rely on assumptions in order to produce a working model and any analysis will only be as good as the validity of these assumptions. For example, assumptions are required to formulate the structure of the model, the costs that are applied beyond the end of the trial, and patient outcomes in the long term. Thus checking the appropriateness of the modelling approach is of importance to ensure that the conclusions drawn from a model-based analysis are valid.

The objective of this systematic review was to examine the published literature that has reported model based economic evaluations that focus on testing and screening for CKD. Its purpose is not to draw conclusions about the cost-effectiveness of different interventions; rather it is to examine how economic models are implemented in this setting. Of particular interest was to gain a greater understanding of how the impact of test accuracy has been incorporated into these analyses, how delays along the testing and diagnostic pathway have been modelled, and whether the impacts of the testing and diagnostic pathway on patients in terms of its effect on quality of life (e.g. anxiety following a positive test) and the costs incurred by patients have been properly considered.

## Methods

### Inclusion Criteria

This study sought to identify model-based economic evaluations that focused on testing for, and diagnosis of, CKD. To be included in this review, studies had to meet the following criteria. They had to focus on albuminuria-based and/or eGFR based testing for CKD and be an economic evaluation, namely CEA, CUA, or CBA that reported an incremental cost-effectiveness ratio (ICER), which is the ratio of the difference between costs and outcomes between the interventions considered in the analysis. Only studies that focused on general, hypertensive and diabetic populations were incorporated, and for studies where the patients were already diagnosed with CKD, only patients with eGFR ≥15 mL/min/1.73m^2^ were considered, with those that focused on patients with kidney failure (eGFR <15 mL/min/1.73m^2^) being excluded from this review. Studies also had to be model-based with those that used costs and outcomes measured from a trial being excluded from this analysis.

### Search Strategy

Articles were identified through searches of electronic databases and hand searching of the bibliographies of the included studies. Studies were limited to those that focused on humans and published in English language. The following databases were searched: Medline (In-Process & Other Non-Indexed Citations and Ovid MEDLINE(R), 1948 to Present), Embase (Embase 1974 to Present) and PsycINFO (1967 to present) accessed via Ovid SP; CINAHL (from 1981 to present) accessed via EBSCO, The NHS Economic Evaluation Database and the HTA database both accessed via the Cochrane Library (Wiley). The search strategy was customized for each database and used a combination of key terms such as “chronic kidney disease”, “cost-effectiveness”, “economic evaluation”, “diagnosis”, “testing”, “proteinuria”, “hypertension”, “diabetes”, “GFR”, “eGFR”, and “microalbuminuria”. Medical Subject Headings (MeSH) terms were applied to each search strategy where appropriate (see S1 Appendix).

### Selection of papers for review

In order to judge the final suitability of studies for inclusion in this review, each study was grouped independently by two of the investigators (A.S. and K.B.) by examining its title and abstract where available, based on the initial criteria described in Table 1. Consensus resolved any differences. The full text was obtained from articles in Groups A-D, and examined to judge their suitability to be included in the review.

**Table 1:** Initial categorization of studies.

*Group*	*Criteria*	*Action*	*Notes*
A	Reported a model-based economic evaluation that incorporates one or more albuminuria- and/or eGFR-based testing strategies targeting the hypertensive, diabetic, or general population	Retrieved full text	
B	Reported an economic evaluation that incorporates one or more albuminuria- and/or eGFR-based testing strategies targeting the hypertensive, diabetic, or general population	Retrieved full text	Unsure if model-based
C	Discussed costs and impacts of one or more testing strategies targeting the hypertensive, diabetic, or general population	Retrieved full text	Unsure if model-based economic evaluation or unsure if focused on albuminuria- or eGFR-based testing
D	Discussed the costs and impact of one or more testing strategies	Retrieved full text	Generally unsure about contents
E	Not relevant	Exclude	E.g. not focused on appropriate testing, or inappropriate patient group, not an economic evaluation

For each paper included in this systematic review, data extraction was conducted in order to answer the following research questions (see S2 Appendix):

- Test accuracy—was the accuracy of each test defined, justified and incorporated in the analysis? How did test accuracy impact on patient outcomes? Were the parameters that define the test accuracy subjected to any sensitivity analysis?
- Modelling approach—what type of model was used (e.g. Markov, decision tree)? How was progression of the chronic nature of CKD described in the analysis?
- Patient outcomes—was the time delay as a result of patients not being placed on the optimum treatment (or management) pathway reflected in the patient outcomes? Did the analysis include the impact on patient outcomes as a result of changing the timing of testing, decision-making and treatment?
- Economic outcomes—were the costs incurred by the patients (societal costs) along the testing pathway incorporated into the analysis

The methodological quality of each paper was assessed through the use of a ten-item checklist [21] (see S3 Appendix). A score was assigned to each paper based on how well it met the criteria; scores of 1.00, 0.50 and 0 were assigned to “yes”, “cannot tell” and “no”, respectively. Thus each paper scored from 0 (bad quality) to 10 (good quality) [22]. The aim of this systematic review was to examine the methods used in describing testing and diagnostic pathways in economic evaluations in this field and not to comment regarding the results and conclusions drawn from these studies. Consequently, no studies were excluded from this review due to issues regarding quality.

## Results

### Study Selection

**Fig 1:**
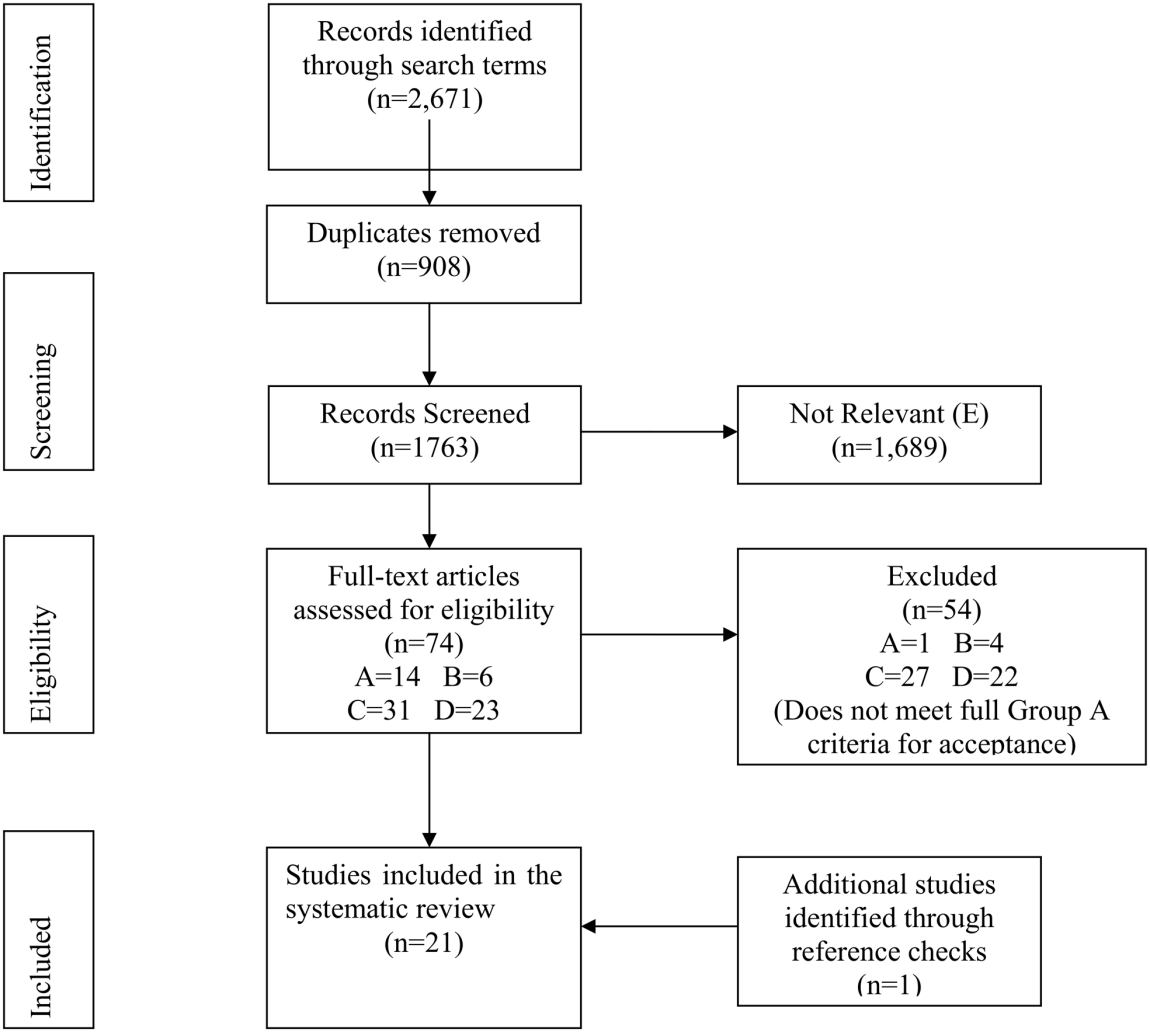
Identification of studies for final review.

The searches were conducted on 17^th^ February 2015; a flow diagram describing the outcome of these searches is shown in Fig 1. The initial search strategy of the databases identified 2,671 studies of which 908 were duplicate studies and 1,689 were excluded because they contained no material of relevance to this study. Hence, 74 studies were selected for full-text review. Overall, 21 studies met the criteria for inclusion in this systematic review (Table 2), including some studies that were originally misclassified as being in Groups B-D based on their title and abstract.

**Table 2:** Characteristics of included studies (UAE- urine albumin excretion, GFR-glomerular filtration rate, UPCR-urine protein to creatinine ratio, UACR-urine albumin to creatinine ratio, LYG-life years gained, QALY-quality adjusted life year, CKD-chronic kidney disease).

Study	Study Population	Method	Type of Testing	Timing	Outcome	Modelling Approach	Perspective	Test accuracy considered
Adarkwah et al (2010) Germany	Newly diagnosed 2 type diabetes Aged 50	albuminuria (UAE 30–300 mg/d); gross albuminuria (UAE >300 mg/d)	Monitoring	Annual	LYG / QALY	Markov	Health care provider	Yes
Adarkwah et al (2011) Netherlands	Newly diagnosed 2 type diabetes Aged 50	albuminuria (UAE 30–300 mg/d); gross albuminuria (UAE >300 mg/d)	Monitoring	Annual	LYG / QALY	Markov	Health care provider	Yes
Boersma et al (2010) Netherlands	General Population Aged 28–75	Albuminuria (UAE 30–300 mg/d)	Screening	One-off	LYG	Markov	Health care provider	Yes
Boulware et al (2003) US	General Population Aged 50	Proteinuria (reagent strip)	Screening	Annual	QALY	Markov	Societal	Yes
Den Hartog et al (2009) US	General Population (Hypothetical) Aged 60	eGFR (<60 ml/min/1.73m^2^); serum creatinine 1.06–1.36 (mg/dl)	Screening	Annual	QALY	Markov	Health care provider	Yes
Farmer et al (2014) UK	Type 1 and Type 2 diabetes patients	UACR (>2.5mg/mmol for men and >3.5mg/mmol for women)	Monitoring	Annual	QALY	Individual based simulation model	Health care provider	Yes
Golan et al (1999) US	Newly diagnosed diabetes aged 50	albuminuria (UAE 30–300 mg/d); gross proteinuria (UAE >300 mg/d)	Monitoring	Annual	LYG / QALY	Markov	Societal	No
Hoerger et al (2010) a & b; US	General Population (US) aged 50–90	Albuminuria (UAE 30–299 mg/d)	Monitoring	1-, 2-, 5-, 10-years	QALY	Micro-simulation Markov	Health care provider	Yes
Hoerger et al (2012) US	African / non-African Americans Aged 50+	Albuminuria (UAE 30–299 mg/d)	Monitoring	1-, 2-, 5-, 10-years	QALY	Micro-simulation Markov	Health care provider	Yes
Howard et al (2010) Australia	Hypertensive / diabetic cohort (Simulated) Aged 50+	Proteinuria (reagent strip followed by spot UACR >20 mg/mg confirmatory test)	Monitoring	Annual	QALY	Markov	Health care provider	Yes
Kessler et al (2012) Switzerland	General Population Aged 50+	Albuminuria (UACR 30–299 mg/g)	Screening	1-, 2-, 5-, 10-years	QALY	Micro-simulation Markov	Health care provider	Yes
Kiberd et al (1995) Canada	Patients with insulin dependent diabetes mellitus for 5 years	Albuminuria (UAE >20 μg/min) or hypertension or macroproteinuria, or both (dipstick >0.3 g/l or positive Albustix confirmed with >300 mg/d or UAE >200 μg/min proteinuria)	Monitoring	Annual	QALY	Markov	Third party and government	Yes
Kiberd et al (1998) Canada	Patients with insulin dependent diabetes mellitus for 5 years	albuminuria (UAE >20 μg/min or UACR 30 mg albumin/g creatinine); macroproteinuria (dipstick >0.3 g/l or positive Albustix confirmed with >300 mg/day or >200 μg/min proteinuria)	Monitoring	Annual	LYG / QALY	Markov	Third party and government	Yes
Kiberd et al (1999) US	Male (Pima Indians) with diabetes at diagnosis	albuminuria (UACR >3 mg albumin per 1 mmol of creatinine or UACR >30 mg per 1g of creatinine)	Monitoring	Annual	LYG	Markov	Third party and government	No
Kondo et al (2012) Japan	General Population Aged 40–74	Proteinuria (reagent strip) eGFR (<50ml/min/1.73m^2^)	Screening	One-off	QALY	Markov	Societal	No
Le Floch et al (1993) France	Fictitious cohort of 10,000 diabetes patients	Albuminuria (UAE >20 μg/min)	Monitoring	Annual	QALY	Markov	Health care provider	Yes
Manns et al (2010) Canada	General Population	eGFR (<60 ml/min/1.73 m^2^)	Screening	One-off	QALY	Markov	Health care provider	No
Palmer et al (2008) US	Type 2 diabetes and hypertension	Albuminuria (UAE 20–199 μg/min)	Monitoring	Annual	QALY	Markov	US third-party health insurance payer	Yes
Sekhar et al (2010) US	School children aged 8–15	Proteinuria (reagent strip)	Screening	One-off	Case of CKD diagnosed	Decision Tree	Health care provider	Yes
Siegel et al (1992) US	Newly diagnosed with diabetes Aged 15	Proteinuria (>300 μg/min)	Monitoring	Annual	LYG	Markov	Health care provider	No
Srisubat et al (2014) Thailand	45 year old patients with diabetes with normotension	Proteinuira (reagent strip)	Monitoring	Annual	QALY	Markov	Societal	Yes

### Characteristics of Selected Studies

Of the studies in this review, 19 considered proteinuria, of which 6 were focused on reagent strip (‘dipstick’) proteinuria testing and 13 on albuminuria. Three studies evaluated eGFR testing (one study incorporating both proteinuria and eGFR). One further study described the parameterisation of a model of CKD without considering an intervention [23], but this was considered in this review since this model was subsequently applied in a number of other studies [24–26]. Eight studies originated from the US, three studies were from Canada and the Netherlands, and one each from Australia, France, Germany, Japan, UK, Switzerland, and Thailand.

The majority of studies focused on adults drawn from the general population [23–31], with one study considering schoolchildren [32]. Ten studies focused on monitoring patients with diabetes [12,33–41] and two studies considered monitoring patients with diabetes and hypertension [42,43]. In all included studies, testing was either administered in primary care or had no stated setting.

The time horizons adopted in these studies were based either on the final age of the patient population or on a specific period of time. Six studies conducted model analysis until the patient population had reached 75 years old or more [24–26,28,30 43]. Three Studies adopted a lifetime time horizon until all the patients had died [31,35,41]. Four Studies adopted a time horizon of 50 years or more [33,34,38,39], Five studies adopted 25–30 years [12,36,37,40,42], and Two studies implemented time horizons of less than 20 years [27,29]. Only one study did not incorporate a time horizon. This study adopted a decision tree approach and considered the cost per case of CKD detected for urine dipsticks targeting school-aged children [32].

### Modelling Issues and Quality

The quality assessment scores applied to each study in this review ranged from 6.5 to 10 (S4 Appendix). The majority of studies had a score of 9 or more (n = 17), but only 2 studies had perfect scores. The mean quality score was 8.7 across all studies and the standard deviation was 0.98. All but three studies [29,36,43] lost one point for not discussing all the issues relevant to users, which in this case meant that the studies did not discuss the impact of testing from the patient perspective (e.g. costs incurred, anxiety, etc.).

Eight studies in this review examined the cross-validity of the model results by comparing them to results obtained from other similar studies [27,30,33,34,36,39,42,43]. Five studies examined both the internal validity of the model and its cross-validity [12,24–26,31]. Eight studies did not examine the validity of the models or their results [28,29,32,35,37,38,40,41].

### How was test accuracy considered in the analysis?

Four studies did not consider the issue of test accuracy at all [30,31,40,41], with the remainder considering the issue to some extent. Only one study which examined serum creatinine versus eGFR explicitly modelled all the different permutations of test status following a test i.e. true positive, true negative, false positive, and false negative [29]. In this case, for patients with a false positive test result, these were assumed to incur the one-time costs of diagnostic workup before being returned to the true negative state after one cycle which in this model was one-year. In this study eGFR reporting was found to be cost-effective compared to serum creatinine. At baseline, the authors assumed that a false positive test result would not have any impact on patient health. However, when this assumption was relaxed during sensitivity analysis with the assumption that a false positive test might impact on QoL for one year, it reversed the conclusions drawn from the model with serum creatinine testing becoming more cost-effective compared to eGFR. Patients in the false negative state were assumed to remain unaware of their disease status, until the next cycle, and the next round of screening one year later, leading to unnecessary disease progression and the possibility of reaching kidney failure before being identified as requiring treatment.

In the other studies where test inaccuracy was considered, eight studies (one testing for eGFR [29]) incorporated its impact on the analysis by including the unnecessary additional costs incurred due to confirmatory tests amongst patients that tested false positive [12,24,26–29,42,43]. However in all cases the possibility of the confirmatory tests also being false positive was not examined and the impacts on patients of the initial positive test (e.g. increased anxiety) were also not incorporated. Where the possibility of a false negative test was incorporated into the analysis, this was implemented by assuming that the patient had to wait one time cycle (one year in all cases) before being offered another test, with the patient thereby experiencing unnecessary disease progression for at least one time cycle [24,28,29,36,37,42].

Finally, the evidence used to inform sensitivity and specificity values was frequently taken from single studies [24,26,32,36,37,42,43], rather than using more robust evidence from meta analyses or a systematic review. Interestingly in the studies by Adarkwah et al [33,38] adopting a specificity of 100% in the base-case analysis was regarded as a conservative approach as ‘treating false positives leads to cost savings’. However confirmatory testing or any negative impacts on the patient of a positive test were not incorporated into the analysis.

### What approach was used in the modelling of disease progression?

The majority of studies used a Markov model approach to model disease progression (see Table 2). Four studies applied a micro-simulation approach to a Markov model structure [23–26]. This means that the model followed patients individually as they passed through different states of the Markov model rather than monitoring populations of patients. One study utilized an individual based model structure in which the timing of a cardiovascular event was estimated in order to estimate lifetime outcomes and the quality-adjusted life expectancy associated with different screening strategies [12]. Only one study did not model disease progression and instead used a decision tree approach to establish the cost-effectiveness of identifying cases of CKD amongst school children [32].

Amongst the studies that described the progression of CKD this was conducted through the use of progressive albuminuria or GFR states. Only 3 studies explicitly allowed for the possibility of the reversibility in CKD severity [12,27,36], In one further study there was no progression between stages of CKD, instead the probabilities of progression to ESRD were based on the renal function stratum of the initial population at time zero [30]. In another, CKD was represented as a single health state [31].

### Were the impacts of any delays on the testing and treatment pathway considered?

Since the majority of the studies incorporated in this review focused on screening, the most common type of delay considered was in getting disease positive patients tested for the first time. The screening studies in this review all considered annual screening [12,24–26,28,29,33,36–43]. A number of studies then extended this screening interval in sensitivity analysis up to 10 years [12,26,28], thereby implying that a patient that becomes eligible may have to wait up to 10 years before diagnosis and treatment is offered. Interestingly no study considered the possibility of a screening interval of less than 1 year. It is noted that screening interventions tend to become more cost-effective for longer screening intervals as under these circumstances it is likely that more true positive individuals that are eligible for treatment will be identified during each screening event (e.g. Hoerger et al (2010), Hoerger et al (2012) and Kessler et al (2012) [24–26]. Moreover there were no studies that considered the possibility of symptomatic patients seeking diagnosis and treatment outside of the defined screening intervals.

No studies considered the possibility of a delay in receiving results following testing. While it is acknowledged that for the modern health care systems considered in this review, these delays will be minimal, delays in the communication of abnormal test results to patients can still occur. In addition, receiving immediate treatment following a confirmed positive test seems to be an implicit assumption made in all of the studies where testing and treatment was considered.

### Were costs incurred by patients (societal costs) along the testing pathway considered?

Four studies reported adopting a societal perspective [28,30,36,41], with the remainder using only a health-care provider perspective. In the case of the study by Kondo et al (2012) [30], only the costs incurred as a result of the costs of the tests, a detailed patient examination, and then various treatments are incorporated in the analysis, while in the study by Boulware et al (2003) [28] direct costs of medical care were considered as well as the indirect costs of loss of income for people disabled as a result of kidney failure. In the case of Srisubat et al (2014) [36], this study considered the costs for patients as a result of albuminuria progression due to the food, travel, and opportunity costs incurred as a result of travelling to receive testing. Golan et al (1999) [41] reported using a societal perspective, but did not report costs in sufficient detail to gain an understanding of how costs incurred by patients might be incorporated into the analysis. Aside from the study by Srisubat et al (2014) [36] there were no studies that considered the costs incurred by patients as a result of travel costs and loss of income as a result of having to attend for testing (and re-testing). Although this was mitigated as in some cases it was stated that testing could be conducted during a regular primary care appointment [28,35].

## Discussion

This systematic review has focused on the approaches used in model-based economic evaluations of testing and diagnosis for CKD. Twenty-one studies were identified and data was extracted from these to gain insights into how test accuracy, delays along the testing and treatment pathway, and the costs incurred by patients were incorporated in the analysis. Test accuracy was considered in 17 of the studies in this review, although its full implications on model results were not always examined. Typically only the possibility of false positive tests was incorporated in the analysis with patients either assumed to undertake further testing to confirm the original test result, or else receive unnecessary management and treatment. In the case of repeated testing, only the costs of the repeat tests incurred by the health care provider were incorporated into the analysis. This approach meant that the impact on the patient of receiving a false positive test result was overlooked, and as a result the costs incurred by patients in terms of travel costs and loss of earnings due to having to return for further testing were usually not incorporated in the analysis, and neither was the impact of the anxiety associated (i.e. potential reduction in quality of life) with having received a positive test. Moreover the failure to incorporate confirmatory testing for initially positive test results in many of these studies is also a concern, since the variability of the test results for patients in this setting (particularly the elderly) may mean that in the absence of confirmatory testing, patients could be given inappropriate treatment.

False positive tests potentially have a large impact on results drawn from models where the prevalence of a disease is very low [44]. Under these circumstances it is much more likely that a positive test will be false and as such false positive results may have a large impact on the conclusions drawn from a model. For CKD, this is likely to be the case only when screening is targeting younger age groups of the general population where the prevalence of CKD is lower. Only one study incorporated the impact of the anxiety associated with a false positive test, and interestingly found that changing this value across reasonable bounds led to changes in the conclusions drawn from the study, with serum creatinine being preferred to eGFR when larger quality of life adjustments for a false positive CKD diagnosis were applied in the model. In the case of false negative test results these were frequently not considered in these analyses, but when they were, it was assumed that patients with disease would progress as normal until the next opportunity to get tested. False negative tests become important when the prevalence of a disease is very high, and then it becomes more likely that a negative test may be false [44].

The main delays on testing and diagnostic pathways that are modelled in this setting are the delays in disease positive individuals getting tested. These delays are modelled by describing the interval between screening events, and in this setting these intervals are always assumed as being one year or longer. Delays due to receiving testing are obviously important in this setting as these can lead to patients experiencing unnecessary progression of their disease; by contra-distinction it is likely to be cost-effective to extend the screening interval when targeting the general population as most of the population will not experience disease progression. Delays due to symptomatic individuals seeking or failing to seek diagnosis were never considered. This would obviously be more relevant for studies that focused on high-risk individuals such as people with diabetes or those suffering hypertension, and might have an impact on the conclusions drawn from the models. Delays in passing on test results to patients, and delays in patients receiving treatment following a positive test result were never considered. The studies in this review were all conducted in developed settings and so delays like this may be less common, but nevertheless these are issues that might be considered more widely.

Four studies considered a societal perspective; with only one of these studies incorporating the costs incurred by patients as a result of having to attend for testing such as those due to travel costs and the potential loss of income. In some settings e.g. UK, the advice given when conducting economic evaluations is that a health-care perspective should be adopted, meaning that the costs incurred by patients would be outside the scope of the analysis. In addition, some of the studies in this review mentioned that the testing could be conducted alongside a prearranged primary care appointment which would minimize the costs incurred by the patients. Nevertheless patient incurred costs may still be significant, particularly in groups with lower disease prevalence where there are more likely to be false positive tests. It could therefore be argued that only using a health care provider perspective in this and many other settings that consider medical testing may be inappropriate for incorporating some of the issues that are important to patients.

In the majority of studies, no possibility of the reversibility of CKD severity was incorporated into the models. However a small proportion of patients (e.g. people with diabetes) with eGFR>60mL/min/1.73m^2^ with stage 1 or 2 CKD do either spontaneously revert to normalbuminuria or do so under the influence of blood pressure (BP) control, specifically RAAS (Renin-angiotensin-aldosterone system) blockade (with ACE-I or ARB). Similarly, in a small proportion of patients (10–15%) with more advanced CKD with severe hypertension of significant albuminuria, stabilisation of BP control, and implementation of RAAS blockage, can lead to moderate improvements in renal function. These improvements need not be sustained, and experience suggests that the majority of patients then decline again, but perhaps at a slower rate.

From an economic perspective, the implications of ignoring these issues are that additional treatment may be assumed for patients that do not need it, leading to extra costs being incorporated into the analysis. Moreover, additional unrealistic disease progression may lead models to predict more deaths and cases of ESRD than is realistic. While it is acknowledged that it is often difficult to obtain parameter values which describe reverse progression, this should be attempted where possible in order to design more realistic models.

This review incorporated extensive searches of a multiple electronic databases which has increased the likelihood of identifying all the relevant papers in this topic area. This review could have been expanded to incorporate non-English studies; while the grey literature, which is academic literature that is not formally published, was also not considered. With the focus on this study being on the methods used in model based economic evaluations, a further limitation of this study is that the inclusion criteria meant that the studies included in this review sought to answer different research questions, meaning that an overall conclusion regarding the results obtained from these studies could not be drawn. To our knowledge this is the first study that has focused on the methods used in economic evaluations of testing and diagnosis in CKD to describe the testing and diagnostic pathway and the factors that are important to patients. Previous reviews in this area have focused on the results obtained from the studies [45] rather than issues related to the testing pathway that may have a significant impact on patients.

This systematic review has shown that many of the issues that are important to patients when undertaking economic evaluations in diagnosis and monitoring for CKD are often not considered. The impact on patients of a false positive test as a result of the additional costs incurred by patients through receiving further confirmatory tests is frequently overlooked. Moreover at least one study in this review has shown that the inclusion of the impact on quality of life for patients that receive false positive tests has the power to change conclusions drawn from the analysis. Of course it cannot be said that this will be the case in all groups of people with CKD, but the fact that it has not been considered in many previous studies is of concern. Linked to the issue of false positive tests is the wider issue of test accuracy. It is worrying that studies have been conducted in this area in which test accuracy has not been considered in the evaluations. Aside from the issues related to false positive tests described above, a false negative test result means that a cost has been incurred by the health care provider (and patient), but has provided no patient benefit.

**Fig 2:**
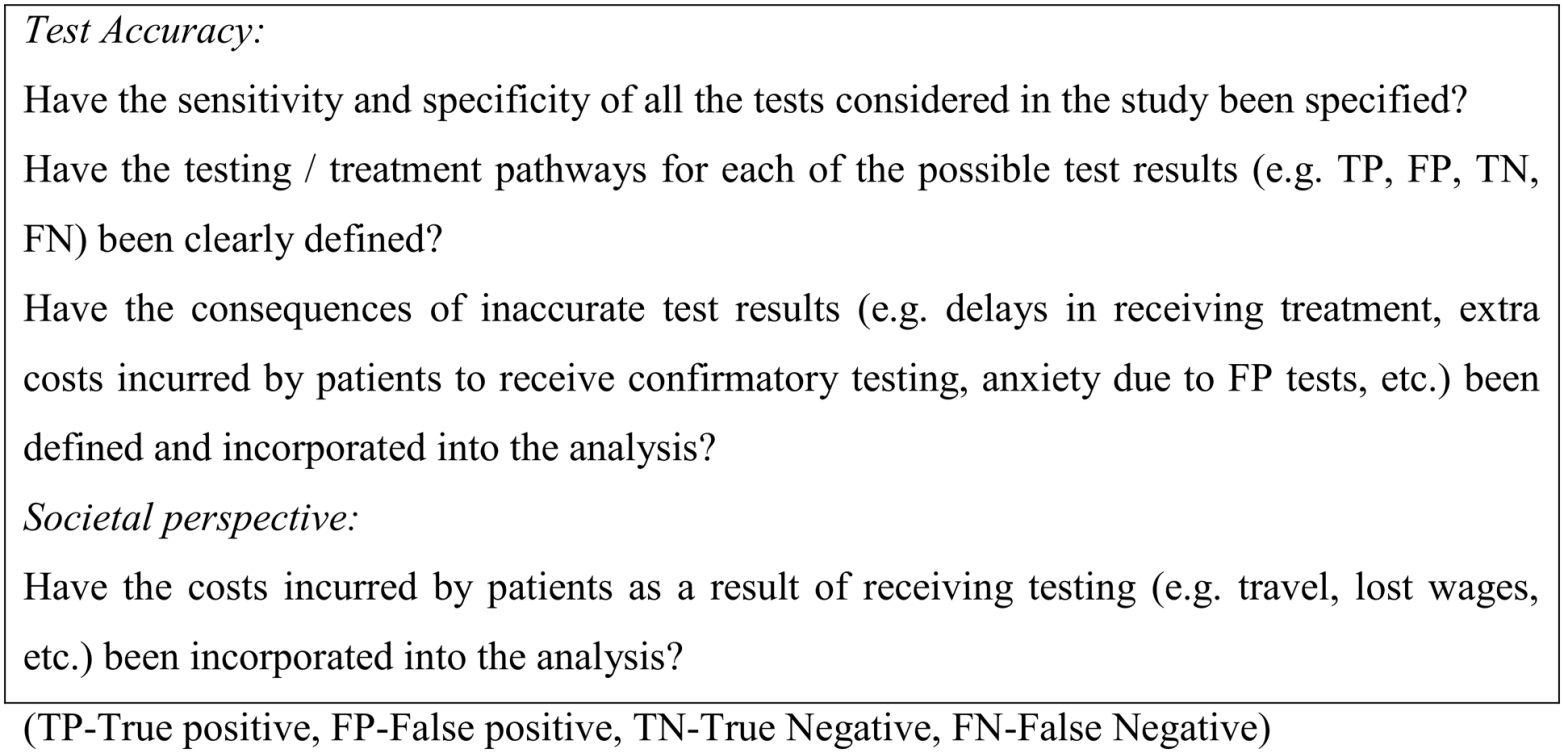
A selection of key questions to be asked when conducting an economic evaluation focused on patient testing (TP-True positive, FP-False positive, TN-True Negative, FN-False Negative).

Future economic evaluations in this area should incorporate test accuracy, and where possible a societal perspective should be adopted. Even if the advice is to use a health care perspective, at the very least a societal perspective should be considered in sensitivity analysis, as it would be important to determine whether this has a significant impact on the conclusions drawn from a model. To ensure these factors are adequately incorporated into an analysis, Fig 2 provides some suggested questions that could be asked when conducting an economic evaluation that incorporates patient testing. While these questions are not definitive, or may not be appropriate for all settings, considering them will ensure that many of the issues that impact on patients are considered in the analysis. At the present time in which patients are becoming ever more involved in setting the research agenda and guiding approaches to research, a societal perspective will become even more important in helping decision makers and patients make informed choices about the use of screening tests.

## Supporting Information

S1 Appendix Search Strategies. (DOCX)

S2 Appendix Data Extraction Form. (DOCX)

S3 Appendix Quality Assessment of Economic Evaluations Table. (DOCX)

S4 Appendix Quality Assessment Scores per Individual Study. (DOCX)
