# Supplementary material for: Methods Used in Economic Evaluations of Chronic Kidney Disease Testing — A Systematic Review
Source: PLoS One. 2015 Oct 14;10(10):e0140063. doi: 10.1371/journal.pone.0140063 (PMC4605841; doi:10.1371/journal.pone.0140063)
Supplement: S1 Appendix — (DOCX) [file pone.0140063.s001.docx]

**Appendix 1: Search Strategies**

Search strategies were modified from those used by The University of York Centres for Reviews and Dissemination to identify economic evaluations.

(http://www.crd.york.ac.uk/crdweb/)

Embase Classic+Embase 1947 to Current (via Ovid)

1. chronic kidney disease/

2. testing.mp.

3. test.mp.

4. 1 or 2 or 3

5. (glomerular filtration rate or gfr or egfr or microalbuminuria or macroalbuminuria or albuminuria or proteinuria or urine albumin electrolyte or UAE or albumin creatinine ratio or ACR or Modification of Diet in Renal Disease or MDRD or dipstick or serum creatinine or Cystatin C or Chronic Kidney Disease Epidemiology Collaboration equation or CKD-EPI).mp. [mp=title, abstract, subject headings, heading word, drug trade name, original title, device manufacturer, drug manufacturer, device trade name, keyword]

6. health economics/

7. exp economic evaluation/

8. exp Health Care Cost/

9. pharmacoeconomics/

10. 6 or 7 or 8 or 9

11. (econom$ or cost or costs or costly or costing or price or prices or pricing or pharmacoeconomic$).ti,ab.

12. (expenditure$ not energy).ti,ab.

13. (value adj2 money).ti,ab.

14. budget$.ti,ab.

15. 11 or 12 or 13 or 14

16. 10 or 15

17. letter.pt.

18. editorial.pt.

19. note.pt.

20. 17 or 18 or 19

21. 16 not 20

22. (metabolic adj cost).ti,ab.

23. ((energy or oxygen) adj cost).ti,ab.

24. ((energy or oxygen) adj expenditure).ti,ab.

25. 22 or 23 or 24

26. 21 not 25

27. animal/

28. exp animal experiment/

29. nonhuman/

30. (rat or rats or mouse or mice or hamster or hamsters or animal or animals or dog or dogs or cat or cats or bovine or sheep).ti,ab,sh.

31. 27 or 28 or 29 or 30

32. exp human/

33. human experiment/

34. 32 or 33

35. 31 not (31 and 34)

36. 26 not 35

37. conference abstract.pt.

38. 36 not 37

39. 4 and 5 and 38

40. limit 39 to English language

*MEDLINE(R) In-Process & Other Non-Indexed Citations and Ovid MEDLINE(R) 1946 to Present (via Ovid)*

1. chronic kidney disease.mp. or Renal Insufficiency, Chronic/

2. Diabetes Mellitus, Type 1/ or diabetes.mp. or Diabetes Mellitus, Type 2/ or Diabetes Mellitus/

3. Hypertension, Renal/ or Hypertension/ or hypertension.mp.

4. 1 or 2 or 3

5. (glomerular filtration rate or gfr or egfr or microalbuminuria or macroalbuminuria or albuminuria or proteinuria or urine albumin electrolyte or UAE or albumin creatinine ratio or ACR or Modification of Diet in Renal Disease or MDRD or dipstick or serum creatinine or Cystatin C or Chronic Kidney Disease Epidemiology Collaboration equation or CKD-EPI).mp. [mp=title, abstract, subject headings, heading word, drug trade name, original title, device manufacturer, drug manufacturer, device trade name, keyword]

6. Economics/

7. exp "costs and cost analysis"/

8. Economics, Dental/

9. exp economics, hospital/

10. Economics, Medical/

11. Economics, Nursing/

12. Economics, Pharmaceutical/

13. (economic$ or cost or costs or costly or costing or price or prices or pricing or pharmacoeconomic$).ti,ab.

14. (expenditure$ not energy).ti,ab.

15. value for money.ti,ab.

16. budget$.ti,ab.

17. 6 or 7 or 8 or 9 or 10 or 11 or 12 or 13 or 14 or 15 or 16

18. ((energy or oxygen) adj cost).ti,ab.

19. (metabolic adj cost).ti,ab.

20. ((energy or oxygen) adj expenditure).ti,ab.

21. 18 or 19 or 20

22. 17 not 21

23. letter.pt.

24. editorial.pt.

25. historical article.pt.

26. 23 or 24 or 25

27. 22 not 26

28. exp animals/ not humans/

29. 27 not 28

30. 4 and 5 and 29

31. limit 30 to english language

*PsycINFO 1806 to Current (via Ovid)*

1. chronic kidney disease.mp.

2. exp Diabetes Insipidus/ or diabetes.mp. or exp Diabetes/ or exp Diabetes Mellitus/

3. exp Hypertension/ or exp Essential Hypertension/ or hypertension.mp.

4. 1 or 2 or 3

5. (glomerular filtration rate or gfr or egfr or microalbuminuria or macroalbuminuria or albuminuria or proteinuria or urine albumin electrolyte or UAE or albumin creatinine ratio or ACR or Modification of Diet in Renal Disease or MDRD or dipstick or serum creatinine or Cystatin C or Chronic Kidney Disease Epidemiology Collaboration equation or CKD-EPI).mp. [mp=title, abstract, subject headings, heading word, drug trade name, original title, device manufacturer, drug manufacturer, device trade name, keyword]

6. "costs and cost analysis"/

7. "Cost Containment"/

8. (economic adj2 evaluation$).ti,ab.

9. (economic adj2 analy$).ti,ab.

10. (economic adj2 (study or studies)).ti,ab.

11. (cost adj2 evaluation$).ti,ab.

12. (cost adj2 analy$).ti,ab.

13. (cost adj2 (study or studies)).ti,ab.

14. (cost adj2 effective$).ti,ab.

15. (cost adj2 benefit$).ti,ab.

16. (cost adj2 utili$).ti,ab.

17. (cost adj2 minimi$).ti,ab.

18. (cost adj2 consequence$).ti,ab.

19. (cost adj2 comparison$).ti,ab.

20. (cost adj2 identificat$).ti,ab.

21. (pharmacoeconomic$ or pharmaco-economic$).ti,ab.

22. 6 or 7 or 8 or 9 or 10 or 11 or 12 or 13 or 14 or 15 or 16 or 17 or 18 or 19 or 20 or 21

23. (task adj2 cost$).ti,ab,id.

24. (switch$ adj2 cost$).ti,ab,id.

25. (metabolic adj cost).ti,ab,id.

26. ((energy or oxygen) adj cost).ti,ab,id.

27. ((energy or oxygen) adj expenditure).ti,ab,id.

28. 23 or 24 or 25 or 26 or 27

29. (animal or animals or rat or rats or mouse or mice or hamster or hamsters or dog or dogs or cat or cats or bovine or sheep or ovine or pig or pigs).ab,ti,id,de.

30. editorial.dt.

31. letter.dt.

32. dissertation abstract.pt.

33. 29 or 30 or 31 or 32

34. 22 not (28 or 33)

35. 4 and 5 and 34

36. limit 35 to english language

*NHS EED (via Cochrane)*

#1 MeSH descriptor: [Renal Insufficiency, Chronic] explode all trees

#2 MeSH descriptor: [Diabetes Mellitus] explode all trees

#3 MeSH descriptor: [Hypertension] explode all trees

#4 glomerular filtration rate or gfr or egfr or microalbuminuria or macroalbuminuria or albuminuria or proteinuria or urine albumin electrolyte or UAE or albumin creatinine ratio or ACR or Modification of Diet in Renal Disease or MDRD or dipstick or serum creatinine or Cystatin C or Chronic Kidney Disease Epidemiology Collaboration equation or CKD-EPI

#5 #1 or #2 or #3

#6 #4 and #5

*CINAHL and Econlit (via EBSCO)*

| S1 | MH “Economics+” | Search modes – Boolean/Phrase |
| --- | --- | --- |
| S2 | MH "Financial Management+" | “” |
| S3 | MH "Financial Support+" | “” |
| S4 | MH "Financing, Organized+" | “” |
| S5 | MH "Business+" | “” |
| S6 | S2 OR S3 OR S4 OR S5 | “” |
| S7 | S1 NOT S6 | “” |
| S8 | MH "Health Resource Allocation" | “” |
| S9 | MH "Health Resource Utilization" | “” |
| S10 | S8 OR S9 | “” |
| S11 | S7 OR S10 | “” |
| S12 | TI (cost or costs or economic* or pharmacoeconomic* or price* or pricing*) OR AB (cost or costs or economic* or pharmacoeconomic* or price* or pricing*) | “” |
| S13 | S11 OR S12 | “” |
| S14 | PT editorial | “” |
| S15 | PT letter | “” |
| S16 | PT commentary | “” |
| S17 | S14 or S15 or S16 | “” |
| S18 | S13 NOT S17 | “” |
| S19 | MH "Animal Studies" | “” |
| S20 | S18 Not S19 | “” |
| S21 | TI glomerular filtration rate or gfr or egfr or microalbuminuria or macroalbuminuria or albuminuria or proteinuria or urine albumin electrolyte or UAE or albumin creatinine ratio or ACR or Modification of Diet in Renal Disease or MDRD or dipstick or serum creatinine or Cystatin C or Chronic Kidney Disease Epidemiology Collaboration equation or CKD-EPI | “” |
| S22 | AB glomerular filtration rate or gfr or egfr or microalbuminuria or macroalbuminuria or albuminuria or proteinuria or urine albumin electrolyte or UAE or albumin creatinine ratio or ACR or Modification of Diet in Renal Disease or MDRD or dipstick or serum creatinine or Cystatin C or Chronic Kidney Disease Epidemiology Collaboration equation or CKD-EPI | “” |
| S23 | S21 or S22 | “” |
| S24 | (MH "Hypertension, Renal+") OR (MH "Hypertension+") OR "hypertension" OR (MH "Masked Hypertension") OR (MH "Hypertension, Renovascular") | “” |
| S25 | (MH "Diabetes Mellitus+") OR "diabetes" OR (MH "Diabetes Mellitus, Type 1+") OR (MH "Diabetes Mellitus, Type 2") | “” |
| S26 | (MH "Renal Insufficiency, Chronic+") OR (MH "Kidney Failure, Chronic+") OR "chronic kidney disease" | “” |
| S27 | S24 or S25 or S26 | “” |
| S28 | LA English | “” |
| S29 | S20 and S23 and S27 and S28 | “” |
