# Supplementary material for: Methods Used in Economic Evaluations of Chronic Kidney Disease Testing — A Systematic Review
Source: PLoS One. 2015 Oct 14;10(10):e0140063. doi: 10.1371/journal.pone.0140063 (PMC4605841; doi:10.1371/journal.pone.0140063)
Supplement: S2 Appendix — (DOCX) [file pone.0140063.s002.docx]

**Appendix II: Data extraction form**

For those papers included in this systematic review, information was extracted using the following form and copied into an Excel spreadsheet.

| **Study information** |
| --- |
| Authors |
| Title |
| Year |
| Location |
| Study Objective |
| Type of testing (e.g. monitoring / screening) |
| Timing of test (e.g. annually, one-off, etc.) |
| Comparators included in analysis |
| Specific patient group |
| Type of economic evaluation |
| Source of data to parameterise model |
| Setting (e.g. resource limited, primary, secondary care) |
| **Modelling methodology** |
| What type of model is used (e.g. Markov model, decision tree)? |
| How is the progression of CKD described in the analysis? |
| What model structures have been used to describe the different model states? |
| Time horizon |
| **Test accuracy** |
| Is test accuracy considered in the analysis and defined and justified? |
| Is the possibility of inaccurate (e.g. TN / FP, incorrect prognosis), indeterminate or test failure considered in the analysis? |
| Is the test accuracy subjected to any sensitivity analysis |
| **Patient outcomes** |
| What patient outcomes are considered in the analysis (clinical events, quality of life, etc.)? |
| Can patient outcomes be influenced by time delay as a result patients not receiving prompt treatment? |
| Can patient outcomes be influenced by the timing of testing, decision making and treatment? |
| **Economic outcomes** |
| Perspective (health care provider, societal) |
| If societal, what societal costs are incorporated in the analysis? |
| What was concluded from the analysis with respect to the cost-effectiveness of the tests? |
