# Supplementary material for: Methods Used in Economic Evaluations of Chronic Kidney Disease Testing — A Systematic Review
Source: PLoS One. 2015 Oct 14;10(10):e0140063. doi: 10.1371/journal.pone.0140063 (PMC4605841; doi:10.1371/journal.pone.0140063)
Supplement: S3 Appendix — (DOCX) [file pone.0140063.s003.docx]

**Appendix III: Quality assessment of economic evaluations**

**Table:** Criteria to assess the quality of economic evaluations (Gonzalez-Perez, 2002)

| Criteria for quality of economic evaluation | |
| --- | --- |
| 1 | Was a well-defined question posed in an answerable form? |
| 2 | Was a comprehensive description of the competing alternatives given? |
| 3 | Was the effectiveness of the programmes of services established? |
| 4 | Were all the important and relevant costs and consequences for each alternative established? |
| 5 | Were costs and consequences measured accurately in appropriate physical units? |
| 6 | Were costs and consequences valued credibly? |
| 7 | Were costs and consequences adjusted for differential timing? |
| 8 | Was an incremental analysis of costs and consequences of alternatives performed? |
| 9 | Was allowance made for uncertainty in the estimates of costs and consequences? |
| 10 | Did the presentation and discussion of study results include all issues of concern to users? |
