# Supplementary material for: Methods Used in Economic Evaluations of Chronic Kidney Disease Testing — A Systematic Review
Source: PLoS One. 2015 Oct 14;10(10):e0140063. doi: 10.1371/journal.pone.0140063 (PMC4605841; doi:10.1371/journal.pone.0140063)
Supplement: S4 Appendix — (DOCX) [file pone.0140063.s004.docx]

## Appendix IV: Quality assessment scores per individual study

|  | Question no (see S3): | | | | | | | | | | |
| --- | --- | --- | --- | --- | --- | --- | --- | --- | --- | --- | --- |
| Study: | 1 | 2 | 3 | 4 | 5 | 6 | 7 | 8 | 9 | 10 | Total |
| Adarkwah et al (2010) | 1 | 1 | 1 | 1 | 1 | 1 | 1 | 1 | 1 | 0 | 9 |
| Adarkwah et al (2011) | 1 | 1 | 1 | 1 | 1 | 1 | 1 | 1 | 1 | 0 | 9 |
| Boersma et al (2010) | 1 | 1 | 1 | 1 | 1 | 1 | 1 | 1 | 1 | 0 | 9 |
| Boulware et al (2003) | 1 | 1 | 1 | 1 | 1 | 1 | 1 | 1 | 1 | 0 | 9 |
| Den Hartog et al (2009) | 1 | 1 | 1 | 1 | 1 | 1 | 1 | 1 | 1 | 1 | 10 |
| Farmer et al (2014) | 1 | 1 | 1 | 1 | 1 | 1 | 1 | 1 | 1 | 0 | 9 |
| Golan et al (1999) | 1 | 1 | 1 | 1 | 1 | 1 | 1 | 1 | 1 | 0 | 9 |
| Hoerger et al (2010b) | 1 | 1 | 1 | 1 | 1 | 1 | 1 | 1 | 1 | 0 | 9 |
| Hoerger et al (2012) | 1 | 1 | 1 | 1 | 1 | 1 | 1 | 1 | 1 | 0 | 9 |
| Howard et al (2010) | 1 | 1 | 1 | 1 | 1 | 1 | 1 | 1 | 1 | 1 | 10 |
| Kessler et al (2012) | 1 | 1 | 1 | 1 | 1 | 1 | 1 | 1 | 1 | 0 | 9 |
| Kiberd et al (1995) | 1 | 1 | 1 | 1 | 1 | 1 | 1 | 1 | 1 | 0 | 9 |
| Kiberd et al (1998) | 1 | 1 | 1 | 1 | 1 | 1 | 1 | 1 | 0.5 | 0 | 8.5 |
| Kiberd et al (1999) | 1 | 1 | 0 | 1 | 1 | 1 | 1 | 1 | 1 | 0 | 8.5 |
| Kondo et al (2012) | 1 | 1 | 1 | 1 | 1 | 1 | 1 | 1 | 1 | 0 | 9 |
| Le Floch et al (1993) | 1 | 1 | 0 | 1 | 1 | 0 | 0 | 1 | 1 | 0 | 6 |
| Manns et al (2010) | 1 | 1 | 1 | 1 | 1 | 1 | 1 | 1 | 1 | 0 | 9 |
| Palmer et al (2008) | 1 | 1 | 1 | 0 | 1 | 1 | 1 | 1 | 1 | 0 | 8 |
| Sekhar et al (2010) | 1 | 1 | 0.5 | 1 | 1 | 1 | 0 | 1 | 0 | 0 | 6.5 |
| Siegel et al (1992) | 1 | 1 | 0.5 | 1 | 1 | 1 | 1 | 1 | 1 | 0 | 8.5 |
| Srisubat et al (2014) | 1 | 1 | 1 | 1 | 1 | 1 | 1 | 1 | 1 | 1 | 10 |
